# Prevalence, Clinical Characteristics and Prognosis of Vascular Disease in Valvular Heart Surgery: A Multi-Centre Study

**DOI:** 10.5334/gh.1462

**Published:** 2025-08-28

**Authors:** Ching-Yan Zhu, Jing-Nan Zhang, Yi-Kei Tse, Qing-Wen Ren, Jia-Yi Huang, Si-Yeung Yu, Ran Guo, Wen-Li Gu, Daniel Tai-Leung Chan, Gregory Y. H. Lip, Kai-Hang Yiu

**Affiliations:** 1 Division of Cardiology, Department of Medicine, The University of Hong Kong Shen Zhen Hospital, Shen Zhen, China; 2 Division of Cardiology, Department of Medicine, University of Hong Kong, Queen Mary Hospital, Hong Kong SAR, China; 3 Division of Cardiothoracic Surgery, Department of Surgery, University of Hong Kong, Queen Mary Hospital, Hong Kong SAR, China; 4 Liverpool Centre for Cardiovascular Science at University of Liverpool, Liverpool John Moores University and Liverpool Heart & Chest Hospital, Liverpool, United Kingdom; 5 Danish Center for Health Services Research, Department of Clinical Medicine, Aalborg University, Aalborg, Denmark

**Keywords:** vascular disease, polyvascular disease, valvular heart surgery

## Abstract

**Background:**

The clinical significance of atherosclerotic disease in more than one vascular bed, that is, polyvascular disease, in valvular heart surgery remains poorly understood. This study aims to establish the prevalence and prognostic value of polyvascular disease for long-term outcomes after valvular heart surgery.

**Methods:**

Patients receiving valvular heart surgery at two tertiary centres from January 1, 2010 to December 31, 2021 were identified. We examined the effect of atherosclerotic disease in three major vascular beds, including coronary artery disease (CAD), ischaemic cerebrovascular accidents (CVA) and peripheral vascular disease (PVD), on postoperative major adverse cardiac events (MACE) and all-cause mortality. Polyvascular disease was defined as atherosclerotic disease in ≥2 vascular beds.

**Results:**

Of 3843 patients (mean age 58 ± 13 years; 52% male), 1266 (33%) had atherosclerotic disease in ≥1 vascular beds, including 207 (5.4%) with polyvascular disease. Patients with vascular disease were older with more comorbidities, higher surgical risk and more aortic stenosis. Over a median follow-up of 6.37 years (IQR: 3.40–9.54), patients with polyvascular disease had the greatest long-term MACE risk [HR: 1.68 (1.35–2.10)], followed by those with monovascular disease [HR: 1.43 (1.24–1.65)]. Both monovascular and polyvascular disease independently predicted mortality and MACE. Patients with extracardiac vascular disease had independently greater long-term MACE risk than CAD [HR: 1.56 (1.27–1.92)].

**Conclusion:**

Patients undergoing valvular heart surgery exhibit a high prevalence of vascular disease. The risk of adverse outcomes rises with both the presence and extent of vascular disease, and extracardiac vascular disease confers greater risk of MACE than CAD.

## Introduction

The global disease burden of valvular heart disease (VHD) is rising along with a worldwide increase in life expectancy and population ageing (1,2). Correspondingly, valvular heart surgery for definitive treatment of VHD has grown in prevalence (3), facilitated by improved access to surgery (4) and expansion of surgical indications (5,6). Postoperative complications, including valve deterioration (7) and heart failure (8), have thus emerged as key determinants of long-term morbidity and mortality. Therefore, novel prognostic factors for long-term risk prediction are imperative for individualised surgical planning and postoperative management, to deliver satisfactory long-term outcomes in this growing cohort.

Atherosclerotic vascular disease, often involving multiple vascular beds, that is, polyvascular disease, is robustly associated with poorer long-term outcomes in large registries and trials (9,10). While not uncommon among patients receiving valvular heart surgery, the prevalence and impact of polyvascular disease on long-term outcomes are poorly characterised. Although surgical risk prediction algorithms incorporating isolated vascular diseases, including the EuroSCORE II and the Society of Thoracic Surgeons (STS) score, may accurately predict operative mortality (11), their accuracy in predicting long-term outcomes after valvular heart surgery beyond the perioperative phase remain modest (12), underscoring the unmet clinical need to incorporate polyvascular disease as an independent entity for accurate long-term risk prediction.

To address these aspects, we aimed to describe the prevalence, clinical characteristics and the prognostic value of vascular disease and polyvascular disease in patients receiving valvular heart surgery.

## Methods

### Study population

From January 2010 to December 2021, 3879 patients who presented for valvular heart surgery at two tertiary referral centres in Hong Kong (Queen Mary Hospital, Queen Elizabeth Hospital) were recruited into the Chinese Valvular Heart Disease Study (CVATS) database and retrospectively analysed. Patients were excluded if clinical, surgical, or follow-up data were incomplete (n = 36). 3843 patients in total were included in the final analysis. This study was part of the Chinese Valvular Heart Disease Study evaluating the epidemiology, pathophysiology and outcomes in Chinese patients with VHD (13), and was approved by the Institutional Review Board of the Hong Kong Hospital Authority (Hong Kong West Cluster).

### Clinical and surgical parameters

Baseline history of comorbidities (diabetes, hypertension, hyperlipidaemia, atrial fibrillation, heart failure and chronic renal disease) was obtained from the Clinical Data Analysis and Reporting System (CDARS), a centralised inter-hospital electronic database, at the time of surgery. Baseline prescription of antihypertensive, lipid-lowering and antiplatelet medications was reviewed, along with preoperative New York Heart Association (NYHA) functional status and laboratory data. Valvular lesions, left ventricular ejection fraction (LVEF) and systolic pulmonary artery pressure (SPAP) were documented during preoperative echocardiography and confirmed during surgery. Surgical risk was evaluated using the logistic EuroSCORE (14).

Vascular disease was defined as the presence of one or more of the following at baseline, based on clinical diagnosis, angiographic findings or intervention need: coronary artery disease (CAD), ischaemic cerebrovascular accidents (CVA; including ischaemic stroke and transient ischaemic attack) and peripheral vascular disease (PVD). Polyvascular disease was defined as vascular disease involving ≥2 vascular beds.

### Outcomes

The primary outcome in this study was postoperative major adverse cardiac events (MACE), defined as the composite of all-cause mortality, heart failure (HF) readmission, myocardial infarction and stroke, diagnosed based on the ninth revision of the International Classification of Diseases (ICD-9). The secondary outcome was all-cause mortality.

### Statistical analysis

Continuous data were presented as mean ± standard deviation, while categorical data were presented as frequencies and percentages. Comparisons between continuous variables were done with the Kruskal–Wallis rank sum test, while Fisher’s exact test or Pearson’s χ^2^ test was used for categorical variables as appropriate. Multivariable Cox proportional hazard regression analyses were performed to assess the association between vascular disease and postoperative MACE and mortality, with four models incrementally adjusting for baseline demographics, comorbidities, medications and surgical risk factors. For postoperative MACE, Fine-Gray analysis was also performed to account for non-cardiovascular death as a competing risk.

Subgroup analyses were also performed according to vascular disease subtype and valve operated. Linear regression was performed to investigate potential collinearities between age and all comorbidities, medications and surgical risk factors.

Risk attenuation analysis was used to determine the extent of risk associated with vascular disease attributable to other risk factors. Percentage attenuation of associations between vascular disease and postoperative outcomes was measured as [β_unadjusted model_ – β_adjusted model_)/β_unadjusted model_] × 100 (%), with β denoting regression coefficients of different adjustment models. 95% confidence intervals (95% CI) around percentage attenuation values were derived from 1000-time bootstrap resampling. Formal risk reclassification analyses were performed by calculating continuous net reclassification improvement (cNRI) for MACE and mortality. Nested Cox regression analysis was used to determine the incremental prognostic value of vascular disease compared to various risk prediction models. All statistical analyses were performed using R version 4.2.1. A two-tailed *p*-value < 0.05 denoted statistical significance.

## Results

### Baseline characteristics

**Figure 1:**
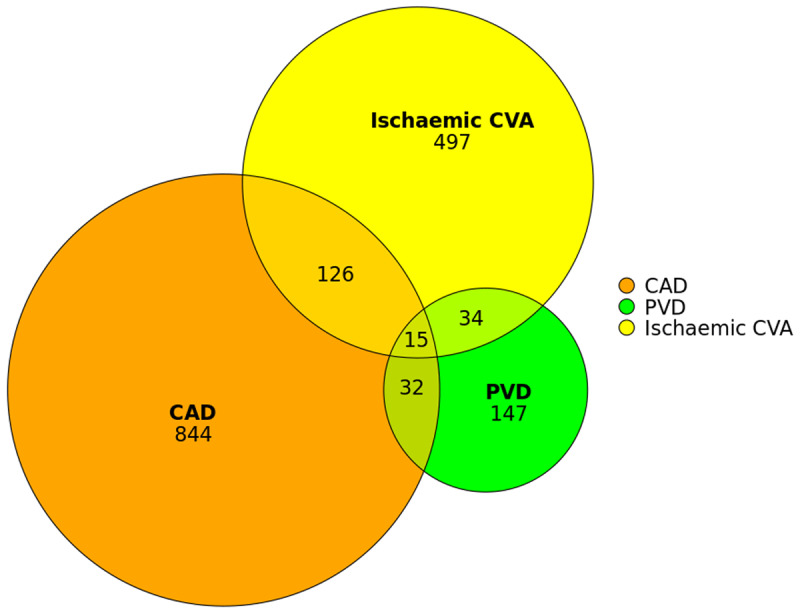
Distribution of vascular disease subtypes. CAD, coronary artery disease; PVD, peripheral vascular disease; CVA, cerebrovascular accident.

Of 3843 patients, 1266 (33%) had vascular disease in ≥1 vascular bed, including 207 (5.4%) with polyvascular disease (16.4% of those with vascular disease). The distribution of different vascular disease subtypes is shown in Figure 1. Baseline clinical characteristics are shown in Table 1. Patients with vascular disease were older and more often male, with higher BMI, smoking rates, cardiovascular comorbidities and medications, with a graded increase from no vascular disease to polyvascular disease.

**Table 1:** Baseline characteristics of patients undergoing valvular heart surgery.


CHARACTERISTIC	OVERALL, *N* = 3843	NO VASCULAR DISEASE, *N* = 2577	MONOVASCULAR DISEASE, *N* = 1059	POLYVASCULAR DISEASE, *N* = 207	*p*-VALUE

Demographic data					

Age (years)	58 (13)	56 (14)	63 (11)	65 (10)	<0.001

Male sex	1997 (52%)	1243 (48%)	616 (58%)	138 (67%)	<0.001

Body mass index, kg/m^2^	23.2 (4.2)	23.1 (4.3)	23.3 (4.0)	24.1 (3.9)	<0.001

Medical characteristics					

Diabetes	361 (9.4%)	144 (5.6%)	160 (15%)	57 (28%)	<0.001

Hypertension	695 (18%)	344 (13%)	268 (25%)	83 (40%)	<0.001

Hyperlipidaemia	321 (8.4%)	128 (5.0%)	147 (14%)	46 (22%)	<0.001

Heart failure	1339 (35%)	823 (32%)	422 (40%)	94 (45%)	<0.001

Atrial fibrillation	1246 (32%)	806 (31%)	361 (34%)	79 (38%)	0.050

Chronic kidney disease	121 (3.1%)	61 (2.4%)	39 (3.7%)	21 (10%)	<0.001

Coronary artery disease	844 (22%)	–	671 (63.4%)	173 (84%)	<0.001

Peripheral vascular disease	147 (3.8%)	–	66 (6.2%)	81 (39%)	<0.001

Ischaemic cerebrovascular accident	497 (13%)	–	322 (30.4%)	175 (85%)	<0.001

Coronary angiography					<0.001

One vessel with >50% stenosis	226 (6.9%)	0 (0%)	181 (19%)	45 (23%)	

Two vessels with >50% stenosis	116 (3.5%)	0 (0%)	87 (8.9%)	29 (15%)	

Three vessels with >50% stenosis	129 (3.9%)	0 (0%)	93 (9.5%)	36 (18%)	

Ankle-brachial index	1.12 (0.12)	1.12 (0.11)	1.10 (0.12)	1.03 (0.18)	0.152

Prior revascularisation	240 (6.2%)	0 (0%)	191 (18%)	49 (24%)	<0.001

Diseased vascular beds					

0	2577 (67%)	2577 (100%)	–	–	

1	1059 (28%)	–	1059 (100%)	–	

2	192 (5.0%)	–	–	192 (93%)	

3	15 (0.4%)	–	–	15 (7.2%)	

Vascular disease type					<0.001

CAD only	671 (53%)	–	671 (63.4%)	–	

PVD only	66 (5.2%)	–	66 (6.2%)	–	

Ischaemic CVA only	322 (25%)	–	322 (30.4%)	–	

CAD + PVD	32 (2.5%)	–	–	32 (15%)	

CAD + ischaemic CVA	126 (10.0%)	–	–	126 (61%)	

PVD + ischaemic CVA	34 (2.7%)	–	–	34 (16%)	

CAD + PVD + ischaemic CVA	15 (1.2%)	–	–	15 (7.2%)	

Medications					

RAAS inhibitors	1813 (47%)	1081 (42%)	590 (56%)	142 (69%)	<0.001

β-blockers	1653 (43%)	1000 (39%)	533 (50%)	120 (58%)	<0.001

Ca channel blockers	1089 (28%)	614 (24%)	382 (36%)	93 (45%)	<0.001

Diuretics	2312 (60%)	1476 (57%)	685 (65%)	151 (73%)	<0.001

Aspirin	427 (11%)	119 (4.6%)	246 (23%)	62 (30%)	<0.001

Clopidogrel	86 (2.2%)	16 (0.6%)	51 (4.8%)	19 (9.2%)	<0.001

Warfarin	1311 (34%)	901 (35%)	344 (32%)	66 (32%)	0.281

Statins	379 (9.9%)	160 (6.2%)	170 (16%)	49 (24%)	<0.001

Laboratory data					

Creatinine, mmol/L	94 (84)	90 (75)	102 (96)	113 (113)	<0.001

Haemoglobin, g/dL	13.10 (2.07)	13.13 (2.09)	13.01 (2.04)	13.14 (1.98)	0.210


### Surgical characteristics

Table 2 summarises surgical risk factors and characteristics according to vascular disease status. Patients with vascular disease had poorer symptomatic status, higher surgical risk and longer operation times, especially those with polyvascular disease. In terms of valvular pathology, the prevalence of aortic stenosis and degenerative disease was highest in patients with polyvascular disease and lowest in those without vascular disease. Congenital lesions and mitral valve pathologies were more common in patients without vascular disease. Compared to those with monovascular disease, patients with polyvascular disease more frequently received concomitant CABG during valvular heart surgery.

**Table 2:** Surgical characteristics in patients undergoing valvular heart surgery.


CHARACTERISTIC	OVERALL, *N* = 3843	NO VASCULAR DISEASE, *N* = 2577	MONOVASCULAR DISEASE, *N* = 1059	POLYVASCULAR DISEASE, *N* = 207	*p*-VALUE

Preoperative assessment					

NYHA functional class					<0.001

1	581 (15%)	413 (16%)	135 (13%)	33 (16%)	

2	1,830 (48%)	1,294 (51%)	459 (44%)	77 (38%)	

3	1,035 (27%)	638 (25%)	330 (31%)	67 (33%)	

4	353 (9.3%)	200 (7.9%)	125 (12%)	28 (14%)	

LVEF, %	55 (12)	56 (11)	53 (13)	51 (15)	<0.001

SPAP, mmHg	41 (21)	41 (23)	41 (16)	36 (14)	0.002

Logistic EuroSCORE	10 (12)	8 (10)	12 (13)	15 (16)	<0.001

Valvular aetiology					

Infective endocarditis	353 (9.2%)	249 (9.7%)	90 (8.5%)	14 (6.8%)	0.252

Degenerative	1,721 (45%)	1,104 (43%)	504 (48%)	113 (55%)	<0.001

Rheumatic	1,136 (30%)	772 (30%)	314 (30%)	50 (24%)	0.212

Congenital	501 (13%)	394 (15%)	93 (8.8%)	14 (6.8%)	<0.001

Surgical characteristics					

Total bypass time (min)	145 (57)	141 (56)	150 (61)	158 (54)	<0.001

Total cross-clamp time (min)	115 (47)	111 (45)	120 (50)	124 (42)	<0.001

Concomitant aortic surgery	292 (7.6%)	192 (7.5%)	77 (7.3%)	23 (11%)	0.144

Concomitant CABG	398 (10%)	0 (0%)	307 (29%)	91 (44%)	<0.001

Primary valvular pathology					

Aortic stenosis	735 (19%)	426 (17%)	246 (23%)	63 (30%)	<0.001

Aortic regurgitation	671 (17%)	453 (18%)	180 (17%)	38 (18%)	0.861

Mixed aortic valve disease	322 (8.4%)	204 (7.9%)	102 (9.6%)	16 (7.7%)	0.223

Mitral stenosis	487 (13%)	331 (13%)	128 (12%)	28 (14%)	0.766

Mitral regurgitation	1,412 (37%)	969 (38%)	378 (36%)	65 (31%)	0.145

Mixed mitral valve disease	365 (9.5%)	247 (9.6%)	106 (10%)	12 (5.8%)	0.162

Valvular surgery type					

Aortic valve surgery	1759 (46%)	1098 (43%)	541 (51%)	120 (58%)	<0.001

Mitral valve surgery	2284 (59%)	1559 (60%)	619 (58%)	106 (51%)	0.024

Multi-valve surgery	1461 (38%)	999 (39%)	397 (37%)	65 (31%)	0.101


CABG, coronary artery bypass graft; LVEF, left ventricular ejection fraction; NYHA, New York Heart Association; SPAP, systolic pulmonary artery pressure.

### Outcomes

During a median follow-up period of 6.37 years (IQR: 3.40–9.54 years), 1176 MACE (23%) [576 (45%) with vascular disease; 600 (23%) without vascular disease, *p* < 0.001] and 713 deaths (19%) [359 (28.4%) with vascular disease; 354 (14%) without vascular disease, *p* < 0.001] were recorded.

**Figure 2:**
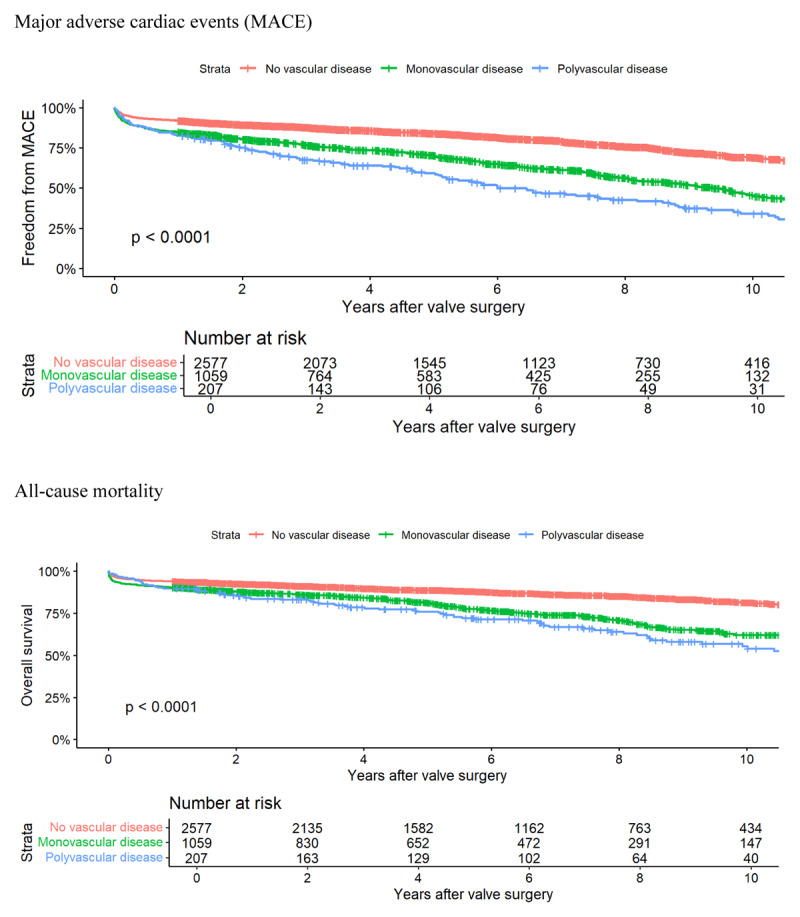
Freedom from postoperative outcomes according to vascular disease status.

Patients with polyvascular disease experienced the highest rates of postoperative MACE and all-cause mortality, followed by patients with monovascular disease and no vascular disease (*p* < 0.001; Figure 2). Postoperative rates of HF readmission, myocardial infarction and stroke were also greatest in those with polyvascular disease (Supplementary Figure 1).

After adjustment for baseline demographics (age, sex) (Model 1), comorbidities (diabetes, hypertension, atrial fibrillation, hyperlipidaemia, heart failure, chronic renal disease and smoking; Model 2), medications (antihypertensives, antiplatelets, statins and warfarin; Model 3) and surgical risk factors (NYHA functional class, LVEF, logistic EuroSCORE, multi-valve surgery, concomitant aortic surgery and prior revascularisation; Model 4), both monovascular disease and polyvascular disease remained independently associated with both postoperative MACE and all-cause mortality (Table 3; Supplementary Table 1 and Supplementary Figure 2).

**Table 3:** Cox proportional hazard regression for postoperative MACE.


VARIABLE	UNIVARIATE HR (95% CI)	MODEL 1 (AGE + SEX ADJUSTED)	MODEL 2 (MODEL 1 + COMORBIDITIES)	MODEL 3 (MODEL 2 + MEDICATIONS)	MODEL 4 (MODEL 3 + SURGICAL RISK FACTORS)

Vascular disease	2.14 (1.91–2.40, *p* < 0.001)	1.69 (1.50–1.91, *p* < 0.001)	1.59 (1.41–1.80, *p* < 0.001)	1.59 (1.40–1.81, *p* < 0.001)	1.47 (1.28–1.68, *p* < 0.001)

Monovascular disease	2.01 (1.78–2.28, *p* < 0.001)	1.60 (1.41–1.82, *p* < 0.001)	1.53 (1.34–1.74, *p* < 0.001)	1.53 (1.34–1.75, *p* < 0.001)	1.43 (1.24–1.65, *p* < 0.001)

Polyvascular disease	2.80 (2.31–3.40, *p* < 0.001)	2.13 (1.75–2.60, *p* < 0.001)	1.92 (1.57–2.35, *p* < 0.001)	1.91 (1.55–2.35, *p* < 0.001)	1.68 (1.35–2.10, *p* < 0.001)

Polyvascular disease (ref. monovascular disease)	1.40 (1.14–1.70, *p* = 0.001)	1.34 (1.10–1.64, *p* = 0.004)	1.29 (1.06–1.58, *p* = 0.013)	1.30 (1.06–1.59, *p* = 0.012)	1.25 (1.01–1.54, *p* = 0.036)


Polyvascular disease was independently associated with a greater risk of MACE than monovascular disease [adjusted HR: 1.25 (1.01–1.54, *p* = 0.036)].

**Figure 3:**
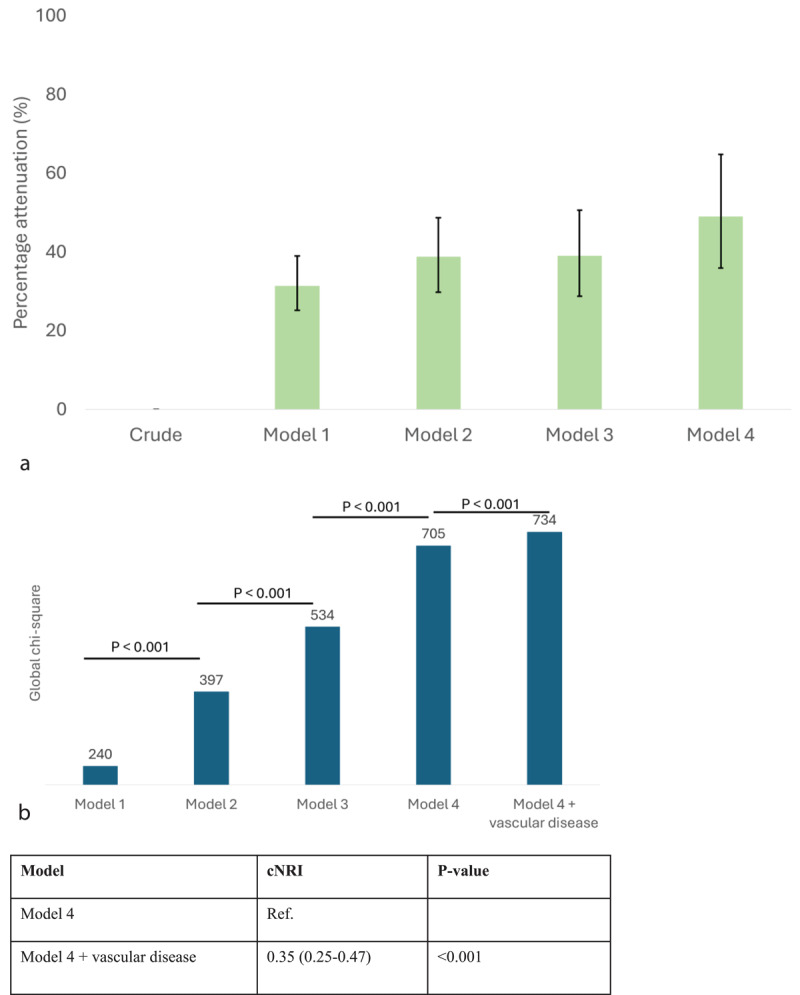
**(a)** Percentage attenuation of MACE risk associated with vascular disease for Models 1 to 4. **(b)** Improvement of prognostic performance and discrimination for MACE after addition of vascular disease to prediction models. cNRI, continuous net reclassification improvement.

The percentage risk attenuation of MACE associated with vascular disease was 31.3%, 38.8%, 39.0% and 49.0% by Models 1–4, respectively (Figure 3a), while the percentage risk attenuation of mortality was 43.2%, 51.1%, 43.2% and 53.6% by Models 1–4, respectively (Supplementary Figure 3a). Adding vascular disease yielded significant reclassification improvement and incremental prognostic value for MACE and all-cause mortality to models combining demographic, clinical and surgical risk factors (Figure 3b; Supplementary Figure 3b). There was no significant collinearity between adjusting risk factors in model development (*R*^2^ = 0.241, adjusted *R*^2^ = 0.236, *p* < 0.001; Supplementary Figure 6), and no significant overfitting was observed when vascular disease was added to Model 4 on calibration (Supplementary Figure 7).

### Subgroup analysis

All three vascular disease subtypes (CAD, ischaemic CVA and PVD) were associated with increased rates of MACE and all-cause mortality (Supplementary Figures 4a and 4b). Subgroup analysis including only patients with vascular disease showed that ischaemic CVA was associated with independently higher rates of MACE (Table 4; Supplementary Figure 5a), while CAD was associated with lower MACE risk (Table 4; Supplementary Figure 5b). Ischaemic CVA and PVD remained independently associated with both MACE and all-cause mortality after adjustment (Supplementary Table 2) and extracardiac vascular disease was associated with greater MACE risk than CAD [adjusted HR: 1.56 (1.27–1.92)].

**Table 4:** Cox proportional hazard regression of MACE according to vascular disease subtype in patients with vascular disease only.


VASCULAR DISEASE SUBTYPE	UNIVARIATE HR (95% CI)	MODEL 1 (AGE + SEX ADJUSTED)	MODEL 2 (MODEL 1 + COMORBIDITIES)	MODEL 3 (MODEL 2 + MEDICATIONS)	MODEL 4 (MODEL 3 + SURGICAL RISK FACTORS)

PVD	1.22 (0.96–1.55, *p* = 0.106)	1.31 (1.03–1.67, *p* = 0.026)	1.34 (1.05–1.70, *p* = 0.019)	1.38 (1.08–1.75, *p* = 0.010)	1.21 (0.93–1.57, *p* = 0.157)

Ischaemic CVA	1.38 (1.17–1.63, *p* < 0.001)	1.53 (1.29–1.81, *p* < 0.001)	1.58 (1.33–1.88, *p* < 0.001)	1.55 (1.30–1.85, *p* < 0.001)	1.61 (1.34–1.94, *p* < 0.001)

CAD	0.84 (0.71–0.99, *p* = 0.043)	0.69 (0.58–0.83, *p* < 0.001)	0.64 (0.53–0.77, *p* < 0.001)	0.64 (0.52–0.77, *p* < 0.001)	0.64 (0.52–0.79, *p* < 0.001)

Extracardiac vascular disease	1.19 (1.01–1.41, *p* = 0.043)	1.45 (1.20–1.72, *p* < 0.001)	1.56 (1.30–1.89, *p* < 0.001)	1.56 (1.30–1.92, *p* < 0.001)	1.56 (1.27–1.92, *p* < 0.001)


PVD, peripheral vascular disease; CVA, cerebrovascular accident; CAD, coronary artery disease.

Subgroup analysis according to valve operated showed independent associations between monovascular disease and both mortality and MACE in aortic and mitral valve surgeries (Supplementary Table 3). Polyvascular disease was associated with MACE in both aortic and mitral valve surgeries.

## Discussion

In this large multi-centre cohort of patients undergoing valvular heart surgery with long-term follow-up data and comprehensive clinical and surgical records, our principal findings are as follows: (1) vascular disease and polyvascular disease are prevalent among patients receiving valvular heart surgery; (2) patients with vascular and polyvascular disease possess distinct patterns of VHD, with greater prevalence of aortic stenosis and (3) vascular disease, especially polyvascular disease, carries a significant burden on postoperative MACE and mortality, significantly underestimated by current risk prediction algorithms.

Polyvascular atherosclerotic disease is commonly seen in patients with cardiovascular disease. Polyvascular disease was found in 14%–18% of participants in various international registries and trials investigating atherothrombotic disease and heart failure (9,15,16). However, its pervasiveness among the growing cohort of patients with VHD remains poorly understood. To this end, the present study demonstrates that both vascular disease and polyvascular disease were as prevalent among valvular heart surgery candidates as in other susceptible populations. These findings underscore the heavy burden of vascular disease in patients with VHD, and the clinical necessity of understanding its influence on postoperative outcomes.

The presence of vascular disease is linked to distinct patterns of valvular pathology, namely degenerative aortic stenosis. Generalised atherosclerosis and aortic stenosis share numerous common risk factors, including age, chronic inflammatory disease (17), atherogenic lipoproteins (18,19) and genetic dysregulation (20). Indeed, the prevalence of polyvascular disease was higher among patients requiring intervention for degenerative aortic stenosis, reaching up to 34% (21). Correspondingly, our study reports that the prevalence of aortic stenosis rose with increasing extent of atherosclerotic vascular disease, portraying aortic stenosis as a characteristic valvular manifestation of advanced atherosclerotic burden. Our findings highlight the necessity to understand the interactions and common therapeutic targets between vascular disease and aortic stenosis.

Atherosclerotic vascular disease remains a major cause of mortality and morbidity among patients with VHD (22), especially extracardiac vascular disease. CAD is a routinely monitored driver of perioperative and postoperative mortality in VHD (4,23,24,25). However, a growing body of literature shows that extracardiac vascular disease, while not regularly monitored under current guidelines (4), may carry a greater impact than CAD on postoperative outcomes. This may be attributed to poorer functional status owing to claudication (26), along with increased vascular resistance impairing cardiac function (27). For instance, cerebral atherosclerosis and peripheral vascular disease both predict greater MACE risk after left-sided valve replacement or repair (26,28). Accordingly, our findings not only demonstrate inferior long-term postoperative outcomes linked with all major subtypes of vascular disease, but also show significantly more postoperative MACE among patients with extracardiac vascular disease. Our findings highlight the key role of extracardiac atherosclerosis in influencing long-term postoperative outcomes in VHD, expanding its prognostic indications beyond its inclusion in current cardiac surgical risk scores including EuroSCORE II, which only reliably predict short-term outcomes (11).

Beyond the presence of vascular disease, the extent of vascular disease, mostly omitted in current risk scores, also confers excess risk of long-term mortality and morbidity in patients, including both vascular and non-vascular adverse events. Various interdependent pathways in polyvascular disease including inflammation, endothelial dysfunction and oxidative imbalance (29) confer poorer short-term postprocedural outcomes in VHD patients with polyvascular disease, as seen in the Optimized Catheter Valvular Intervention-TAVI registry (OCEAN-TAVI) (30). Investigating long-term outcomes, our study shows a clear and graded rise in long-term postoperative MACE across patients with increasingly generalised vascular disease. Notably, patients with polyvascular disease not only experienced more vascular MACE, such as myocardial infarction and stroke, but also had more heart failure readmissions than those with less generalised vascular disease, consistent with findings from previous trials (10,31,32). Our findings showcase how polyvascular disease amplifies the risk of both vascular and non-vascular adverse events in patients with VHD compared to monovascular disease, and highlight the importance of incorporating polyvascular disease as an accurate independent marker of long-term postoperative adverse outcomes.

### Clinical implications

Surgical risk prediction scores such as EuroSCORE II and STS score are widely used to estimate short-term morbidity and mortality after valvular heart surgery (11). However, numerous studies observe notable underestimation of long-term mortality (33), especially in those undergoing valvular heart surgery (34). Moreover, while both scores include vascular disease as a predictor of operative risk, the additive risk posed by polyvascular disease is not well defined in either model, and their utility in predicting long-term postoperative outcomes remains suboptimal (34). With population ageing and continued expansion of surgical indications, more robust risk stratification is necessary to cater to growing patient demands and increasing prevalence of polyvascular disease.

Vascular disease, prevalently seen yet often overlooked in valvular heart surgery, confers risk largely uncaptured by current risk prediction algorithms and substantially improves prediction of long-term postoperative outcomes in this underrepresented population. In particular, our study demonstrates the compounding effect of polyvascular disease compared to monovascular disease on long-term postoperative outcomes, an aspect largely omitted in current risk scores. Our findings suggest that identifying occult vascular and polyvascular disease before valvular heart surgery may enable more accurate long-term risk stratification, which may inform more proactive and diverse anti-atherosclerotic strategies, including preoperative statin therapy (35), exercise and prehabilitation (36,37).

### Limitations

This was a retrospective cohort study with flaws and biases inherent to study design. The presence of vascular disease may have been influenced by demographic confounders or medical confounders as well as insufficient screening, despite extensive efforts to adjust for such factors in multivariable analysis. Limited patients with vascular disease were on antiplatelet or lipid-lowering medications at baseline likely from widespread intolerance of adverse effects in our locality (38,39), which may introduce unmeasured confounding and overestimation of its impact on postoperative outcomes despite comprehensive adjustment. Owing to the study period, there was insufficient data documenting predicted mortality with newer surgical risk scores such as EuroSCORE II. Nevertheless, predictive accuracy was similar between logistic EuroSCORE and EuroSCORE II (11). Patients in this study were mostly Asian, and validation in cohorts of other ethnicities or with different burdens of VHD is warranted, although the prognostic significance of vascular disease persisted across various subgroups in this study.

## Conclusion

In this large multi-centre cohort of patients undergoing valvular heart surgery, vascular disease and polyvascular disease were highly prevalent, and were associated with more frequent aortic stenosis. The risk of adverse outcomes rises with both the presence and extent of vascular disease, and extracardiac vascular disease also confers greater risk of adverse events compared to CAD.

## Additional File

The additional file for this article can be found as follows:

10.5334/gh.1462.s1 Supplementary File. Supplementary Tables 1–3, Supplementary Figures 1–7.

## Abbreviations

CAD: coronary artery disease

CVA: cerebrovascular accident

LVEF: left ventricular ejection fraction

MACE: major adverse cardiac events

NYHA: New York Heart Association

PVD: peripheral vascular disease

VHD: valvular heart disease
