# Supplementary material for: Prevalence, Clinical Characteristics and Prognosis of Vascular Disease in Valvular Heart Surgery: A Multi-Centre Study
Source: Glob Heart. 2025 Aug 28;20(1):71. doi: 10.5334/gh.1462 (PMC12396194; doi:10.5334/gh.1462)

Prevalence, clinical characteristics and prognosis of vascular disease in valvular heart surgery: a multi-centre study

Supplementary tables and figures

Legends

Supplementary table 1. Cox proportional hazard regression of all-cause mortality according to vascular disease status

Supplementary table 2. Cox proportional hazard regression of postoperative outcomes according to vascular disease subtype

Supplementary table 3. Adjusted Cox proportional hazard regression of postoperative outcomes according to valve operated

Supplementary figure 1. Postoperative MACE subcomponents in patients with vascular disease

Supplementary figure 2. Postoperative outcomes in patients with vascular disease after adjustment for demographic confounders, comorbidities, medications, and surgical risk factors.

Supplementary figure 3a. Percentage attenuation of all-cause mortality risk associated with vascular disease for Models 1 to 4

Supplementary figure 3b. Improvement of prognostic performance and discrimination for all-cause mortality after addition of vascular disease to prediction models.

Supplementary figure 4a. Postoperative MACE in vascular disease subtypes

Supplementary figure 4b. Postoperative all-cause mortality in vascular disease subtypes

Supplementary figure 5a. Postoperative MACE in patients with ischaemic CVA versus non-ischaemic CVA vascular disease

Supplementary figure 5b. Postoperative MACE in patients with CAD versus extracardiac vascular disease

Supplementary figure 6. Correlation coefficients between demographics, comorbidities, medications, and surgical risk factors

Supplementary figure 7. Calibration plot for predictive models including baseline demographics, comorbidities, medications, surgical risk factors, and vascular disease status on postoperative MACE

Supplementary table 1. Cox proportional hazard regression of all-cause mortality according to vascular disease status

Variable	Univariate HR (95% CI)	Model 1 (age+sex adjusted)	Model 2 (Model 1 + comorbidities)	Model 3 (Model 2 + medications)	Model 4 (Model 3 + surgical risk factors)
Vascular disease	2.13 (1.84-2.47, $p<0.001$)	1.54 (1.32-1.79, $p<0.001$)	1.43 (1.22-1.67, $p<0.001$)	1.52 (1.29-1.79, $p<0.001$)	1.42 (1.19-1.70, $p<0.001$)
Monovascular disease	2.02 (1.73-2.37, $p<0.001$)	1.48 (1.26-1.74, $p<0.001$)	1.41 (1.20-1.67, $p<0.001$)	1.50 (1.27-1.78, $p<0.001$)	1.41 (1.18-1.69, $p<0.001$)
Polyvascular disease	2.65 (2.07-3.38, $p<0.001$)	1.79 (1.39-2.30, $p<0.001$)	1.49 (1.15-1.94, $p=0.003$)	1.62 (1.24-2.12, $p<0.001$)	1.47 (1.11-1.96, $p=0.007$)

Supplementary table 2. Cox proportional hazard regression of postoperative outcomes according to vascular disease subtype

Major adverse cardiac events (MACE)

Vascular disease subtype	Univariate HR (95% CI)	Model 1 (age+sex adjusted)	Model 2 (Model 1 + comorbidities)	Model 3 (Model 2 + medications)	Model 4 (Model 3 + surgical risk factors)
PVD	1.97 (1.56-2.47, p<0.001)	1.79 (1.42-2.25, p<0.001)	1.76 (1.40-2.22, p<0.001)	1.79 (1.42-2.26, p<0.001)	1.41 (1.09-1.82, p=0.009)
Ischaemic CVA	2.16 (1.88-2.47, p<0.001)	1.91 (1.67-2.19, p<0.001)	1.85 (1.61-2.12, p<0.001)	1.82 (1.59-2.10, p<0.001)	1.75 (1.51-2.02, p<0.001)
CAD	1.70 (1.51-1.93, p<0.001)	1.24 (1.09-1.42, p=0.001)	1.13 (0.99-1.30, p=0.078)	1.07 (0.93-1.25, p=0.347)	0.99 (0.84-1.16, p=0.869)

All-cause mortality

Vascular disease subtype	Univariate HR (95% CI)	Model 1 (age+sex adjusted)	Model 2 (Model 1 + comorbidities)	Model 3 (Model 2 + medications)	Model 4 (Model 3 + surgical risk factors)
PVD	2.05 (1.55-2.72, p<0.001)	1.82 (1.37-2.42, p<0.001)	1.72 (1.30-2.29, p<0.001)	1.81 (1.36-2.40, p<0.001)	1.44 (1.04-2.00, p=0.028)
Ischaemic CVA	1.79 (1.50-2.14, p<0.001)	1.53 (1.28-1.83, p<0.001)	1.45 (1.21-1.74, p<0.001)	1.42 (1.19-1.71, p<0.001)	1.30 (1.07-1.58, p=0.008)
CAD	1.88 (1.61-2.20, p<0.001)	1.24 (1.05-1.47, p=0.011)	1.10 (0.92-1.30, p=0.289)	1.19 (0.99-1.44, p=0.067)	1.20 (0.98-1.46, p=0.074)

Supplementary table 3. Adjusted Cox proportional hazard regression of MACE and all-cause mortality according to valve operated

**MACE**	Monovascular disease HR (95% CI)	Polyvascular disease HR (95% CI)
Aortic valve surgery	1.39 (1.14-1.71, p=0.001)	1.69 (1.26-2.27, p<0.001)
Mitral valve surgery	1.57 (1.30-1.90, p<0.001)	1.61 (1.18-2.20, p=0.003)
**All-cause mortality**	Monovascular disease HR (95% CI)	Polyvascular disease HR (95% CI)
Aortic valve surgery	1.42 (1.11-1.82, p=0.006)	1.57 (1.09-2.26, p=0.014)
Mitral valve surgery	1.54 (1.20-1.98, p=0.001)	1.47 (0.96-2.24, p=0.073)

Supplementary figure 1. Postoperative MACE subcomponents in patients with vascular disease

Heart failure readmissions (HFR)

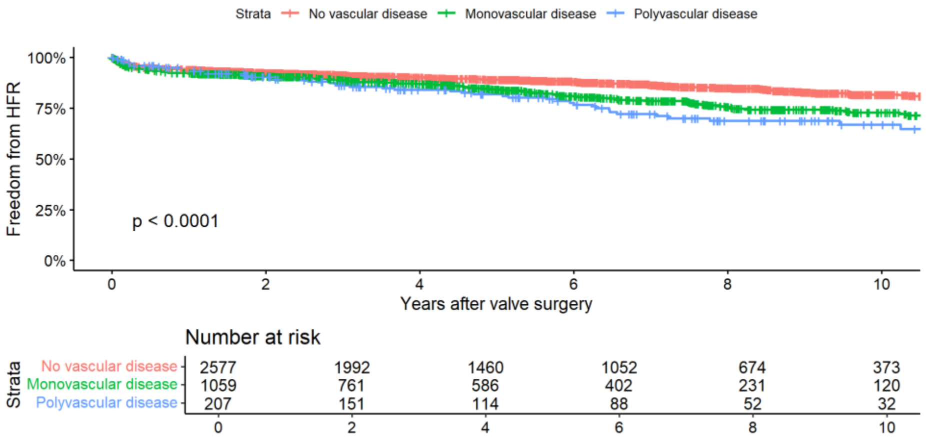

Myocardial infarction

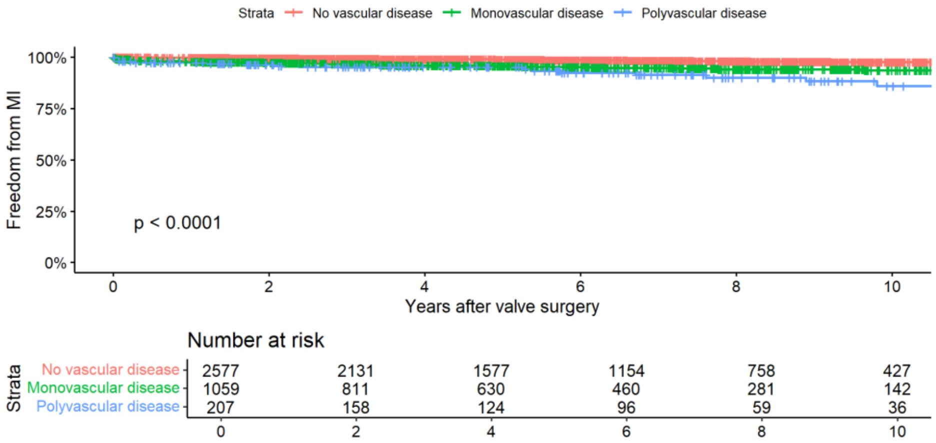

Postoperative stroke

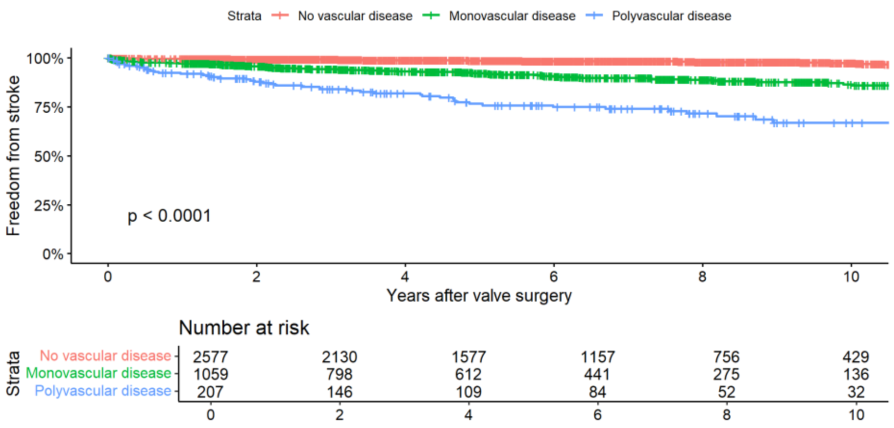

Supplementary figure 2. Postoperative outcomes in patients with vascular disease after adjustment for demographic confounders, comorbidities, medications, and surgical risk factors.

MACE

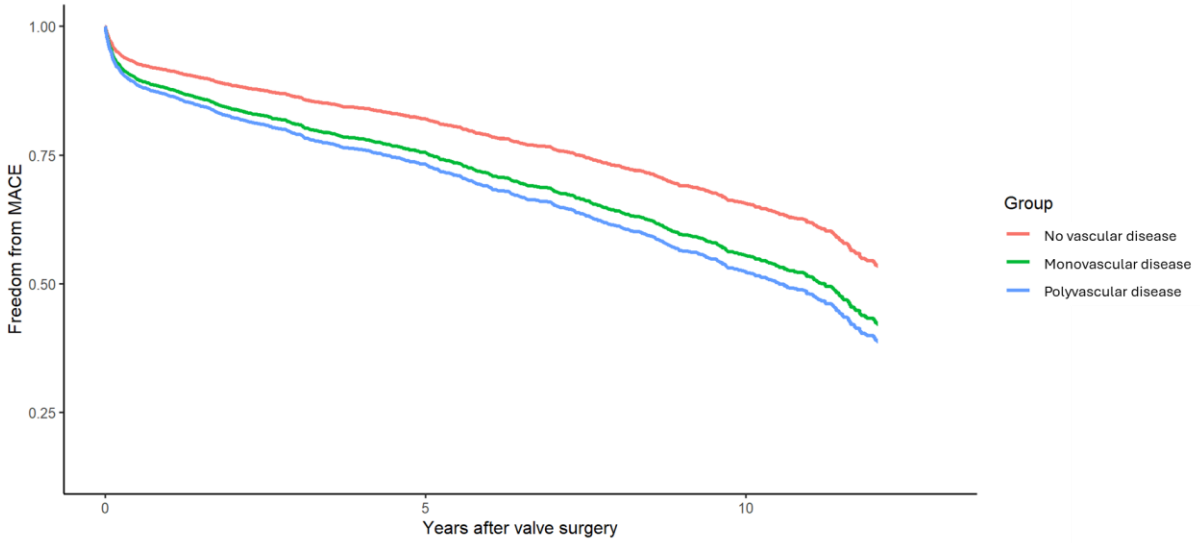

All-cause mortality

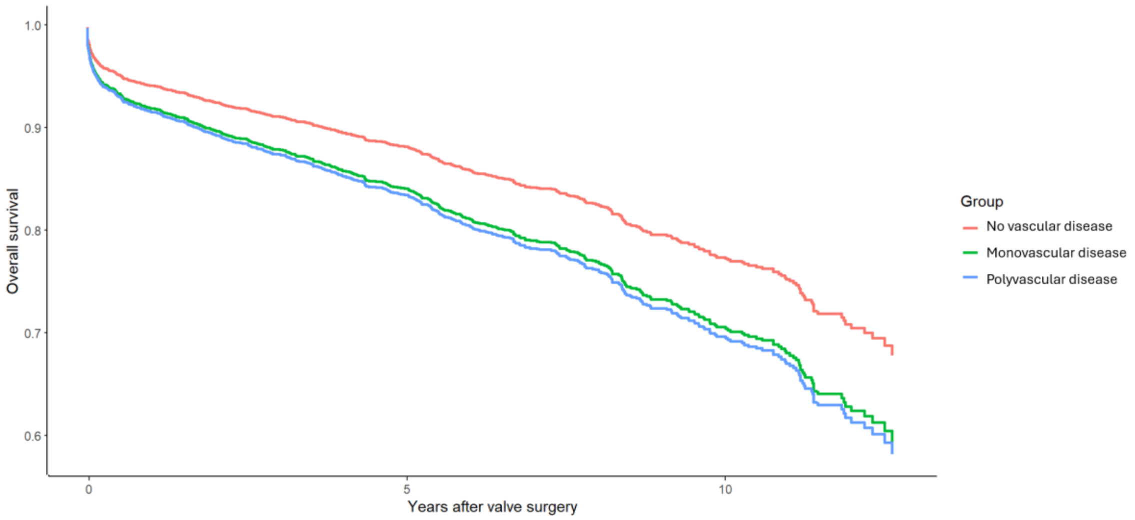

Supplementary figure 3a. Percentage attenuation of all-cause mortality risk associated with vascular disease for Models 1 to 4

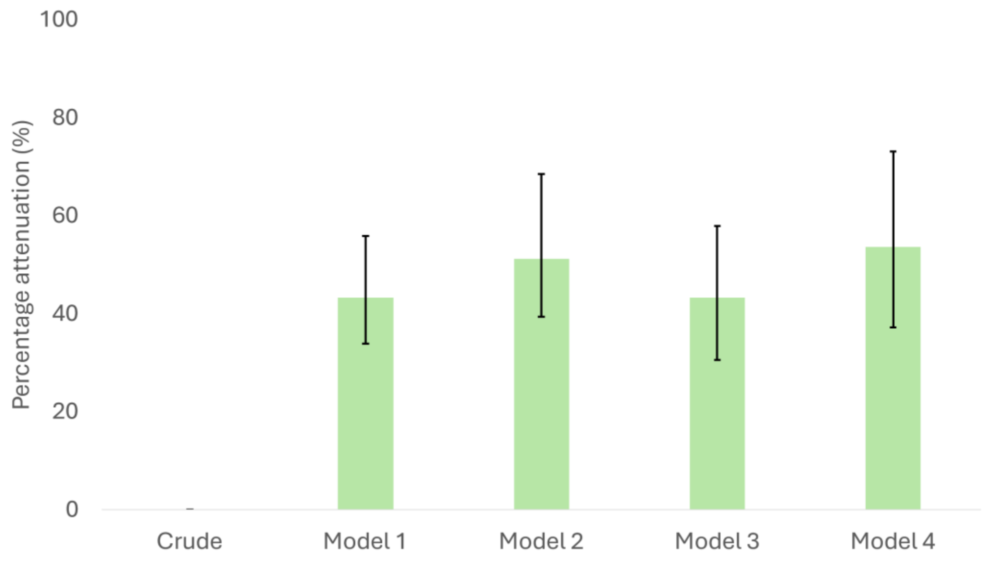

Supplementary figure 3b. Improvement of prognostic performance and discrimination for all-cause mortality after addition of vascular disease to prediction models.

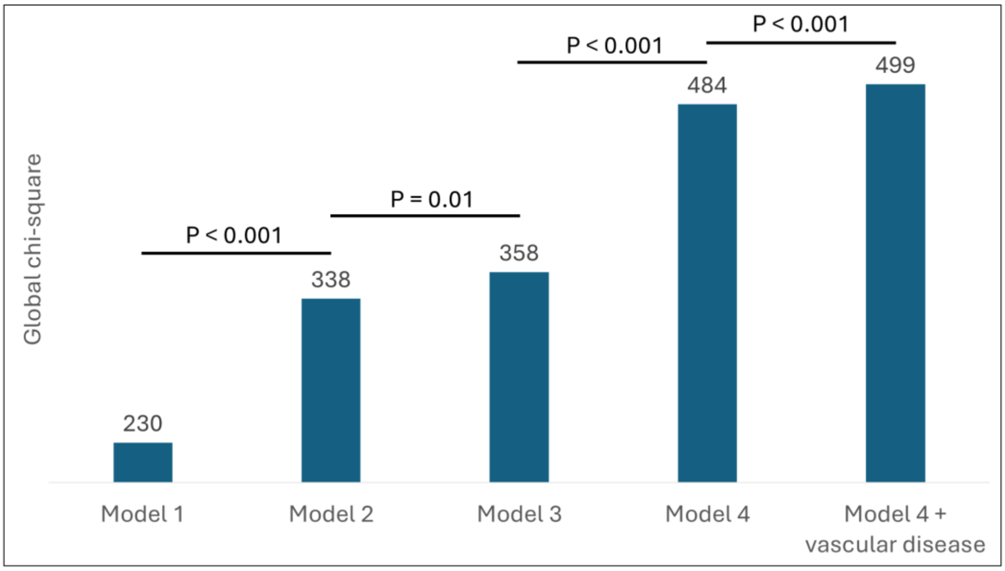

**Model**	**cNRI**	**P-value**
Model 4	Ref.	
Model 4 + vascular disease	0.38 (0.26-0.49)	<0.001

cNRI, continuous net reclassification improvement.

Supplementary figure 4a. Postoperative MACE in vascular disease subtypes

Coronary artery disease (CAD)

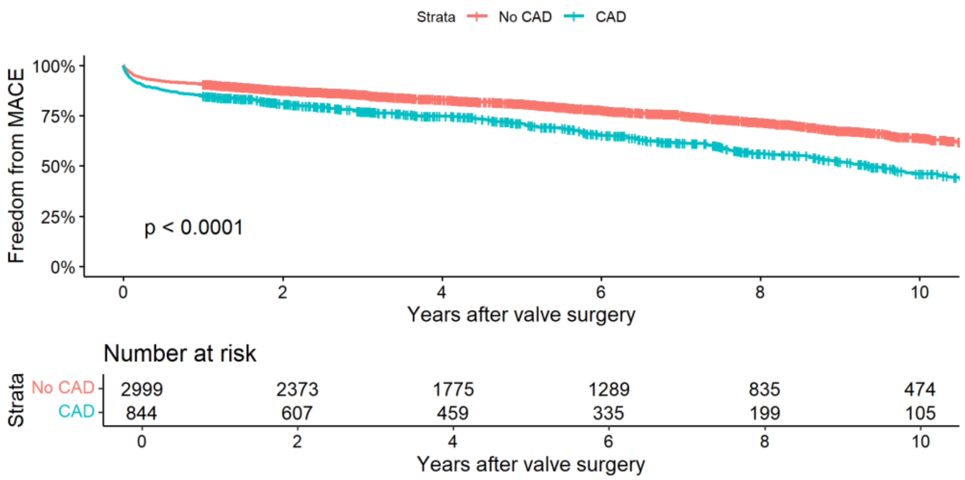

Ischaemic cerebrovascular accident (ischaemic CVA)

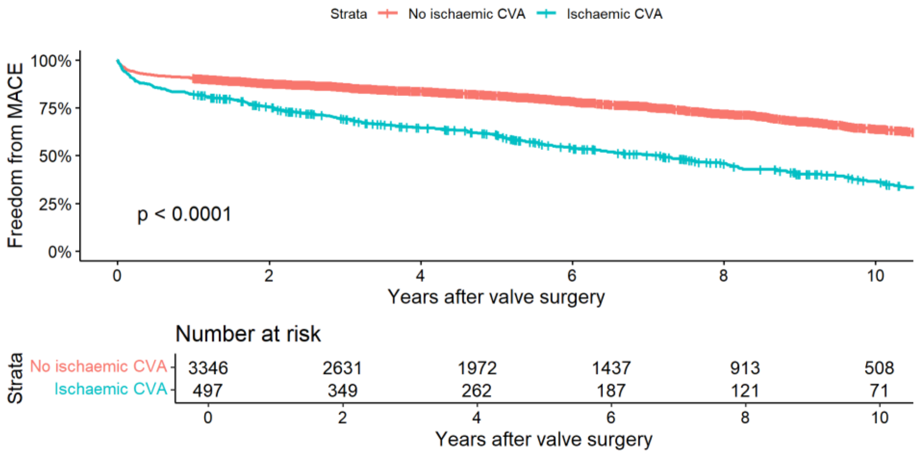

Peripheral vascular disease (PVD)

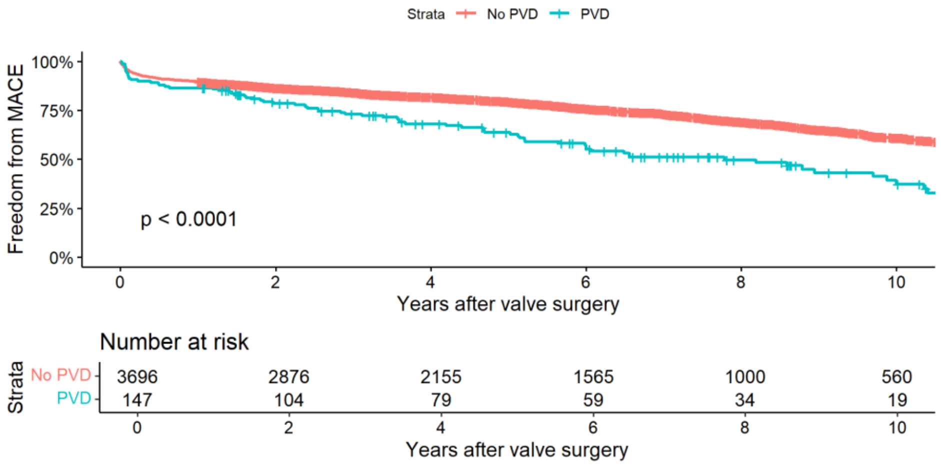

Supplementary figure 4b. Postoperative all-cause mortality in vascular disease subtypes

Coronary artery disease (CAD)

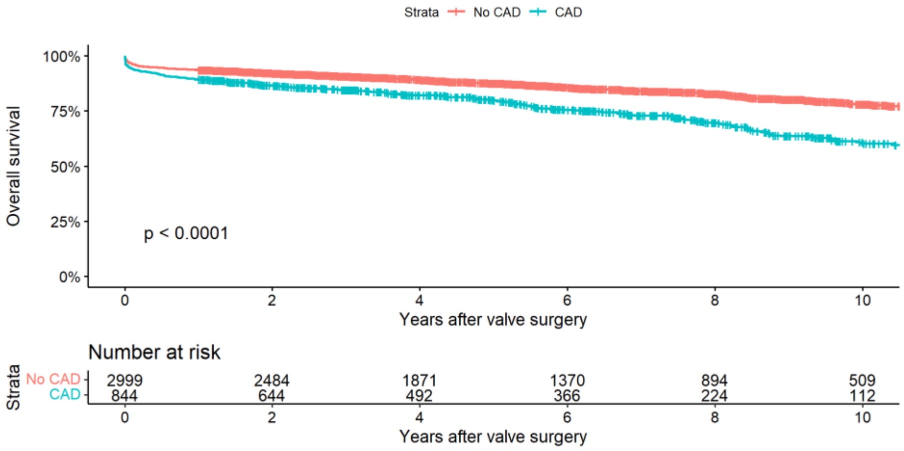

Ischaemic cerebrovascular accident (ischaemic CVA)

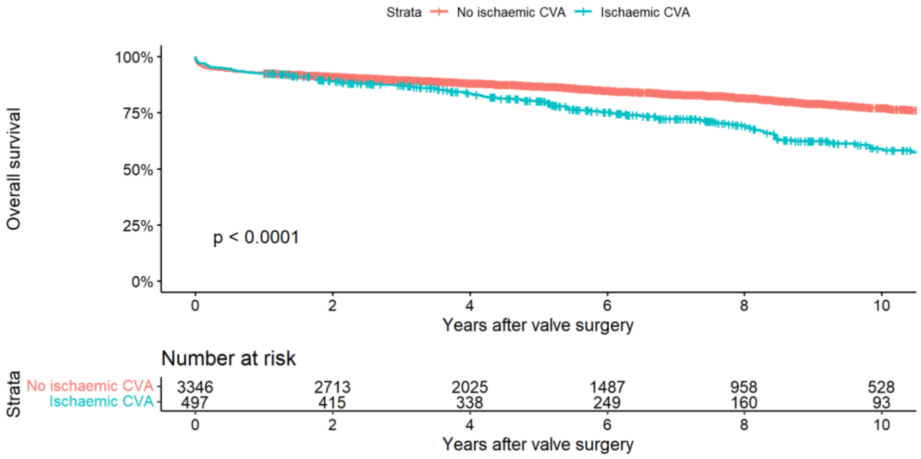

Peripheral vascular disease (PVD)

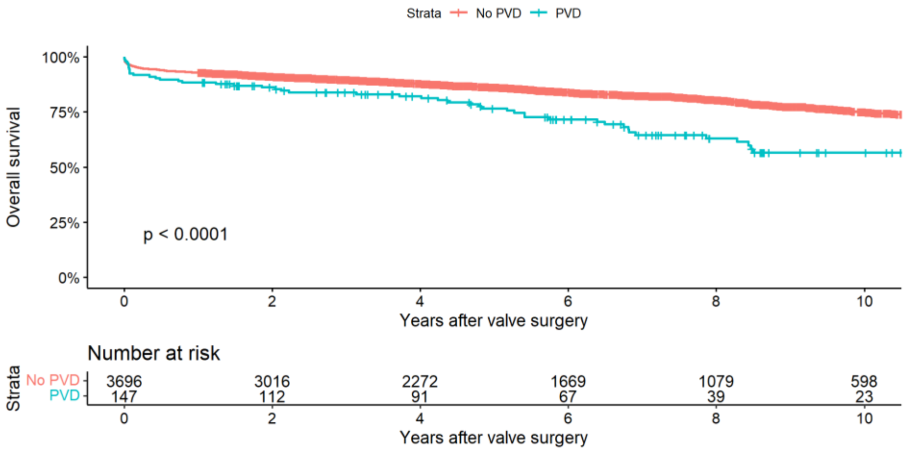

Supplementary figure 5a. Postoperative MACE in patients with ischaemic CVA versus non-ischaemic CVA vascular disease

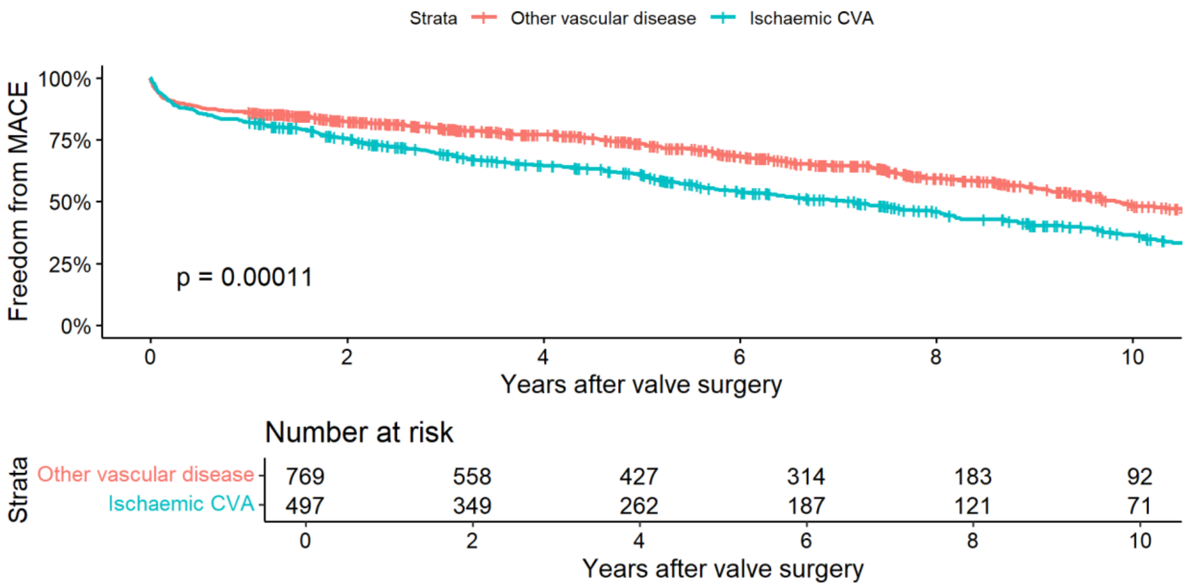

Supplementary figure 5b. Postoperative MACE in patients with CAD versus extracardiac vascular disease

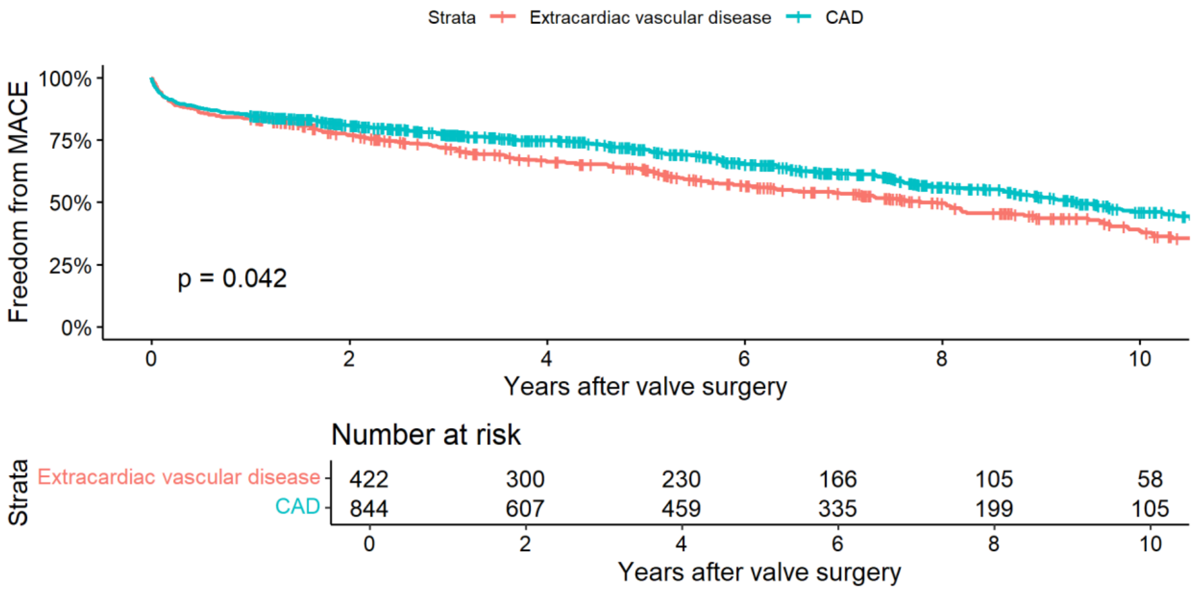

Supplementary figure 6. Correlation coefficients between demographics, comorbidities, medications, and surgical risk factors

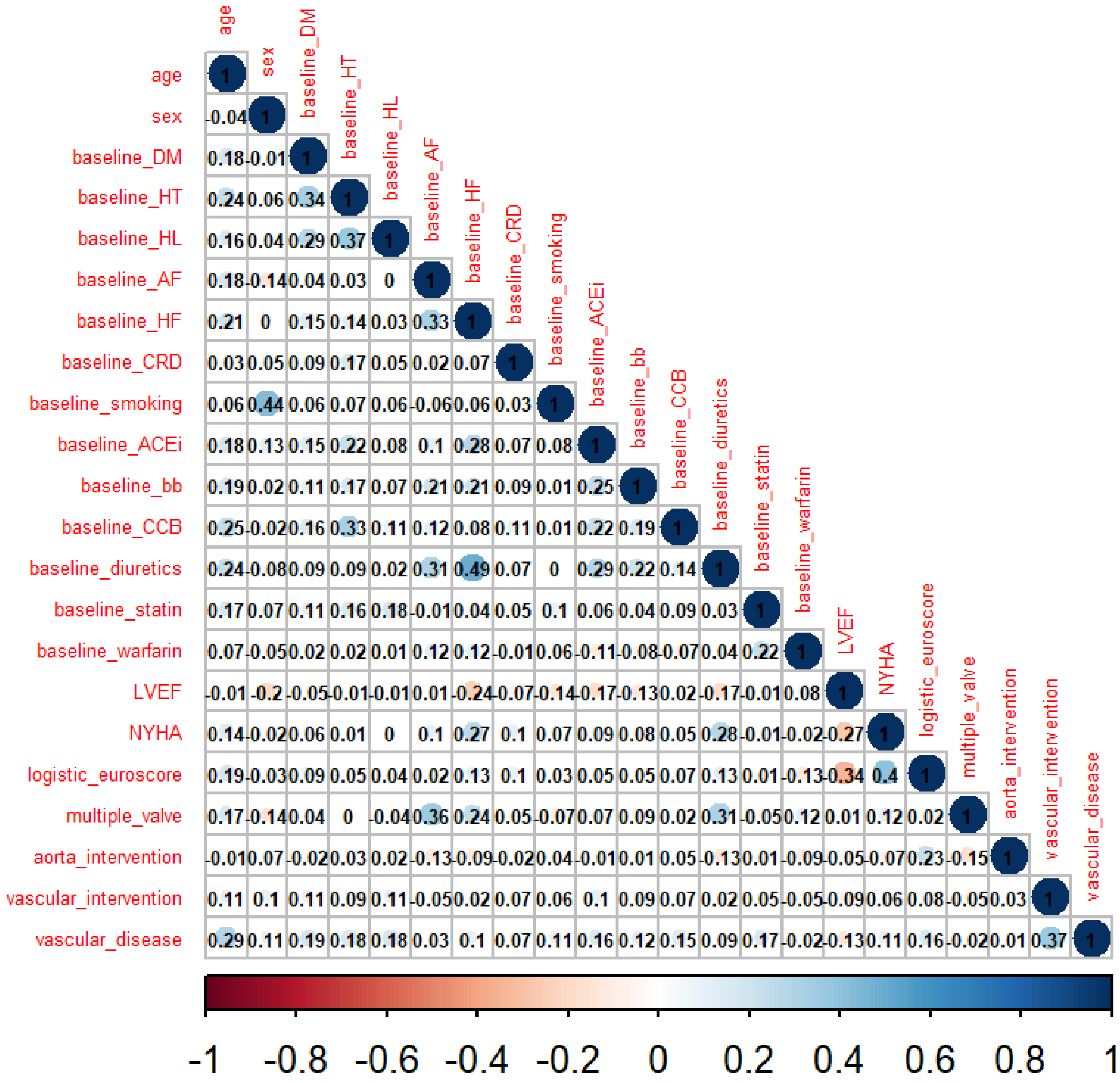

Supplementary figure 7. Calibration plot for predictive models including baseline demographics, comorbidities, medications, surgical risk factors, and vascular disease status on postoperative MACE

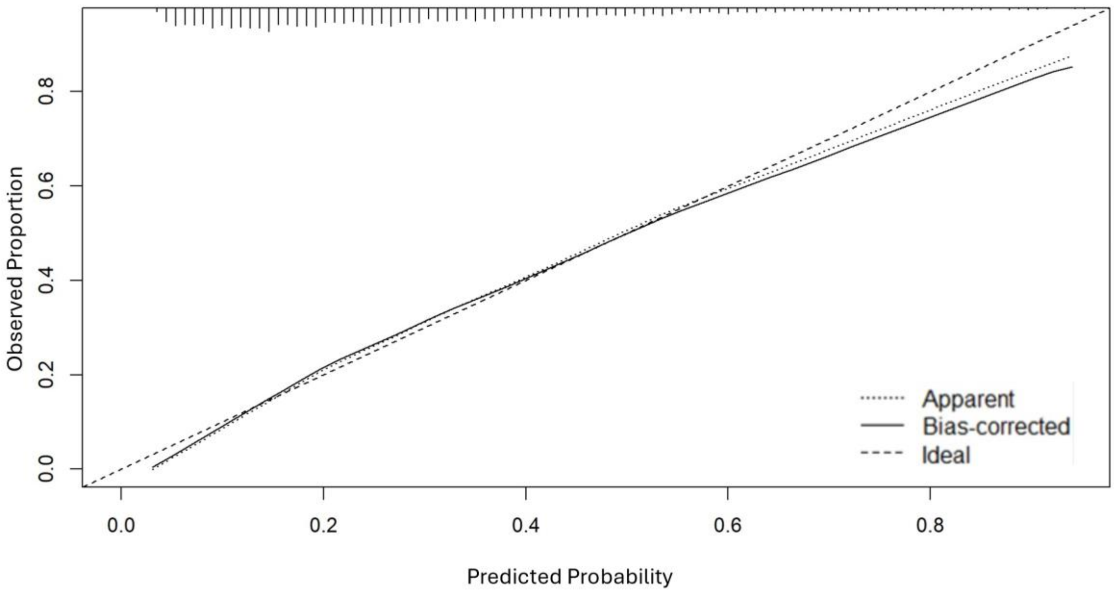

Supplement: Supplementary File. — Supplementary Tables 1–3, Supplementary Figures 1–7. [file gh-20-1-1462-s1.pdf]
